# Promoting Comprehensive Sexuality Education in Pakistan Using a Cocreated Social Media Intervention: Development and Pilot Testing Study

**DOI:** 10.2196/52651

**Published:** 2024-12-20

**Authors:** Furqan Ahmed, Ghufran Ahmad, Katharina Eisinger, Muhammad Asad Khan, Tilman Brand

**Affiliations:** 1 Department of Prevention and Evaluation, Leibniz Institute of Prevention Research and Epidemiology-BIPS Bremen Germany; 2 University of Bremen Health Sciences Bremen Bremen Germany; 3 Cardiff Business School, Cardiff University Cardiff, Wales United Kingdom; 4 Humboldt Park Health Chicago, IL United States

**Keywords:** digital health interventions, sexuality education, social media, influencer marketing, community readiness

## Abstract

**Background:**

Comprehensive sexuality education (CSE) is a curriculum-based approach to learning and teaching about sexuality that focuses on the cognitive, emotional, physical, and social domains. The United Nations Educational, Scientific, and Cultural Organization (UNESCO) CSE guideline emphasizes gender issues and is firmly rooted in a human rights–based approach to sexuality. A recent cross-sectional community readiness assessment in Islamabad, Pakistan, found that the community is at the denial or resistant stage when it comes to implementing school-based sexuality education. The reluctance was attributed to a lack of understanding and widespread misconceptions about CSE.

**Objective:**

This study aims to use the cocreation process to develop, pilot, and evaluate an intervention based on community readiness level to respond to community resistance by introducing CSE content, its anticipated benefits, and addressing prevalent misconceptions through awareness and promotion content for digital social media platforms.

**Methods:**

For the development of the intervention (audio-video content), focus group discussion sessions with key stakeholders were held. Two videos were created in partnership with social media influencers and subsequently shared on Facebook, YouTube, and Instagram. A comprehensive process and performance evaluation of the videos and intervention development phase was conducted to evaluate audience exposure, reach, engagement, demographics, retention, and in-depth insights. The videos were uploaded to social media platforms in June and July 2021, and the data used to assess their performance was obtained in February 2022.

**Results:**

With a total reach (number of people who have contact with the videos) of 432,457 and 735,563 for the first and second videos, respectively, on all social media platforms, we concluded that social media platforms provide an opportunity to communicate, promote, and engage with important stakeholders to raise awareness and obtain support for CSE. According to the findings, the public is responsive to CSE promotion content developed for social media platforms, with a total engagement (the number of people who participate in creating, sharing, and using the content) of 11,578. The findings revealed that male viewers predominated across all social media platforms. Punjab province had the largest audience share on Instagram (51.9% for the first video, 52.7% for the second) and Facebook (44.3% for the first video and 48.4% for the second). YouTube had the highest audience retention, with viewers watching an average of 151 seconds (45%) of the first video and 163 seconds (38%) of the second. With a net sentiment score of 0.83 (minimum=−3, maximum=5), end-user participation was also positive, and audience feedback highlighted the reasons for positive and negative criticism.

**Conclusions:**

To promote sexuality education in Pakistan, it is vital to overcome opposition through sensitizing the society, and digital social media platforms offer a unique, though underused, chance to do so through reliable influencer marketing.

## Introduction

### Background

The International Conference on Population and Development recommended adolescent sexual and reproductive health (SRH) education and promotion in 1994 [1]. Unfortunately, progress has been slow since then due to misunderstandings, organized community opposition to sexuality education, and implementation challenges in many parts of the world [2,3]. According to a 2014 study by the United Nations Educational, Scientific, and Cultural Organization (UNESCO), there are few examples of scaled-up intervention programs addressing sexuality education [4,5]. Comprehensive sexuality education (CSE) is a curriculum-based approach to teaching and learning that focuses on the cognitive, emotional, physical, and social domains of sexuality, according to the UNESCO’s updated technical guidance on CSE [6,7]. Previous CSE guidelines were developed in response to, or focusing on, HIV prevention. However, current findings from research and practice attest to the importance of CSE for the healthy development and general well-being of children and adolescents [6,7]. As a result, the updated guidelines expands on core principles and addresses issues such as early pregnancy, unsafe abortion, and gender-based violence, with a focus on prevention [6,7]. The updated UNESCO technical guidelines place a higher emphasis on gender issues and a firm grounding in a human rights–based approach to sexuality, that is, sexuality is a natural aspect of human development, and it supports organized learning in an age and developmentally-appropriate manner [6,7]. The guidance also emphasizes various sustainable development goals (SDG), particularly those targeted at attaining good health and well-being (SDG 3), quality education (SDG 4), and gender equality (SDG 5) [6-8]. Furthermore, schools provide a setting in which CSE may be introduced at a young age and sequentially across the years using a spiral approach to content development [6,7,9]. Schools also provide an active infrastructure, which includes teachers who are seen as dependable and reliable sources of information by parents, as well as possibilities for long-term programming [6,7,9]. As a result, schools are seen to play a critical role in the delivery of CSE, and the incremental approach is centered on 4 age groups: 5 to <8 years, 8 to <12 years, 12 to <15 years, and 15 to >18 years [6,7,9].

Pakistan presents a challenge to implement and promote reproductive health, women’s empowerment, and sexuality education [10,11]. It is taboo to discuss adolescent SRH and there is a widespread belief, as in many other countries, that exposure to sexuality education may lead to undesirable behaviors [10,11]. Adolescents rely on untrustworthy sources of information such as peers, social media, the internet, and magazines due to a lack of formal sexuality education [12-15]. According to a study conducted in Karachi, Pakistan, such information may lead to mental health problems in adolescents, in addition to exposing them to danger, misinformation, mistreatment, and exploitation [12]. Early marriage is common and there is little recognition that young girls need to be taught about their sexuality and reproductive health rights [16,17]. A key impediment to advancement in these areas is the absence of SRH education in schools and its exclusion from the official educational curriculum [10,18]. Pakistan has a considerable adolescent population, and preventing early marriage, high fertility rates among adolescent girls, and sexual violence against children and adolescents is a challenge [19].

A recent cross-sectional community readiness assessment in Islamabad, Pakistan, concluded that the community is at the denial or resistant stage when it comes to implementing school-based CSE [20]. The reluctance was attributed to a lack of understanding and widespread misconceptions about CSE [20]. Obstacles to school-level implementation are a fraction of the challenges that hinder CSE program implementation [20]. Other obstacles must be addressed, such as policy-level planning, engagement of leaders, community mobilization and support, and financial allocation. An enabling environment that comprises legislative, community, and infrastructure support is crucial for the efficient implementation of CSE [20]. According to the findings, current community support is largely passive, and CSE is primarily debated on digital or social media platforms [20]. Social media may be used as a medium for disseminating knowledge and rebranding a topic that is deemed taboo in society by engaging social media influencers and carefully constructing a narrative around the importance of CSE [20,21]. A possible strategy was thought to be increasing community acceptability and readiness by addressing misconceptions and misunderstandings with counternarratives and scientific evidence [20,21]. Violence (particularly gender-based violence), staying safe, and age appropriateness are some of the major components of CSE that should be stressed for increasing community support [20]. As a response, social media platforms might be used to disseminate a counternarrative and increase community support for implementing CSE in schools.

### Social Media and Health Promotion

Social media platforms, with >3.6 billion active users globally, present significant potential for promoting health, including sexual health [22,23]. Despite this potential, research on the benefits and limitations of social media for health communication remains limited, with most studies being exploratory and descriptive. Social media is frequently used to promote interaction, share health information, and engage people in health communication, offering several advantages such as enhanced information dissemination, increased accessibility, peer support, and public health surveillance [22-24]. Importantly, social media has also been used to raise awareness and advocate against sexual harassment and violence, with evidence of its positive impact on sexual health continuing to grow [22-24]. However, while these platforms are a promising tool for sexual health promotion, the evidence base must be expanded with studies that explore theoretical frameworks and use rigorous methodologies to further establish the effectiveness of these interventions.

Despite the opportunities social media offers for sexuality education, there are notable challenges to consider. One of the main concerns is the unregulated nature of content, which can lead to the spread of misinformation—particularly dangerous when dealing with sensitive topics like sexual health [22-24]. Ensuring that the information shared on social media is accurate, reliable, and easy to understand is critical to realizing the full potential of these platforms. Integrating media literacy into sexuality education programs could be a valuable solution, empowering young people to critically assess the sexual content they encounter online. In tandem with efforts to monitor and improve the reliability of sexual health information on social media, media literacy could enhance the effectiveness of sexuality education delivered through these channels [22-25]. However, further research is essential to identify best practices for using social media in sexuality education and to address the evolving challenges posed by this rapidly changing digital landscape.

There are almost 169 million cellular subscribers in Pakistan, 85 million 3G and 4G mobile technology users, and 87 million internet users [26]. Pakistan has a growing number of internet users and population penetration, making it a favorable setting for digital health interventions, as it is globally ranked tenth in the number of online users [20]. In conservative contexts, digital platforms may be used to engage stakeholders, particularly gatekeepers and influencers. Hence, the purpose of this study was to evaluate the performance (ie, audience engagement and perception) of implementation of an intervention based on community readiness level, which introduces CSE content, addresses prevalent misconceptions, and promotes awareness through social media platforms using the cocreation process.

## Methods

### Cocreation of Complex Health Interventions

More tailored solutions and interventions focused on individuals’ needs and circumstances may be generated in cooperation with careful stakeholder input throughout the development of health interventions [27,28]. Cocreation, as the process is known, has shown significant potential for increased end-user participation and uptake [27,28]. After evaluating a variety of case studies that addressed various health behaviors in diverse groups of people, the principles or steps for cocreating health interventions were developed by Leask et al [27,28]. The following are the four steps of the cocreation process [27]:

1. Planning—What is the goal of cocreation and who should be involved?
2. Implementation—What activities can be used during the process and how can stakeholders commit?
3. Evaluation—How can the cocreation process and outcome be assessed and evaluated?
4. Reporting—How to report the results of the study?

**Figure 1:**
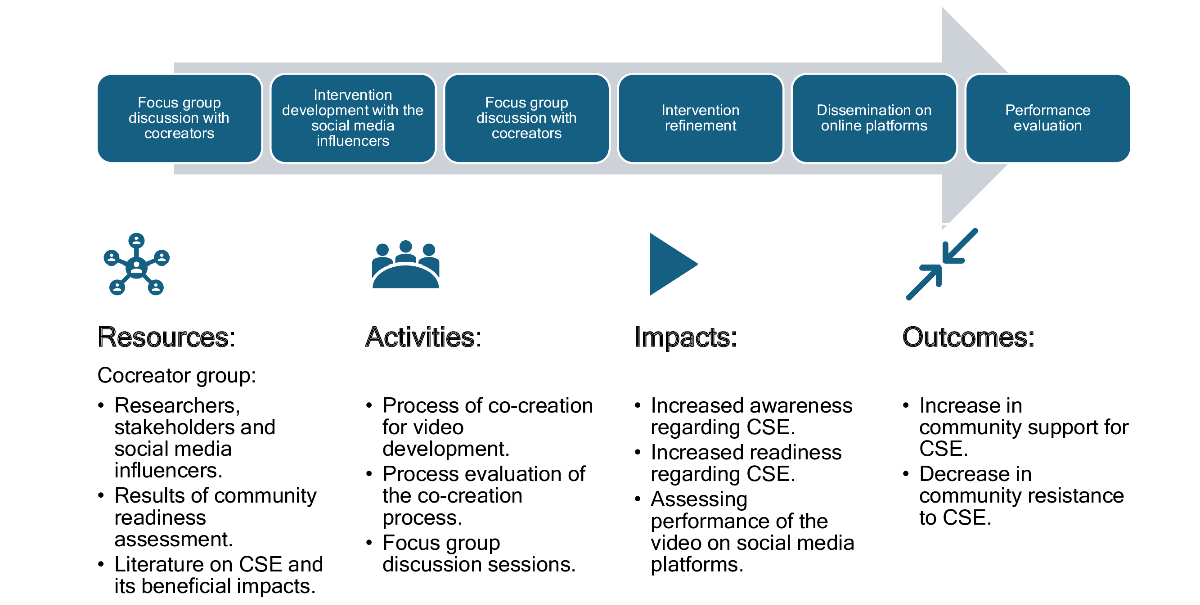
Logic model for the cocreation process, based on the protocol by Leask et al [27] and the Medical Research Council Framework, highlighting resources, cocreation activities, impact, and outcomes. CSE: comprehensive sexuality education.

The intervention for this study was developed with equal engagement from a diverse group of stakeholders using the cocreation concept or process for developing complex health interventions guided by the framework developed by Leask et al [27] for conducting and documenting the process of cocreating complex health interventions. The objective and the reporting framework that were used to develop the intervention are presented in Textbox 1 and Table 1 [27]. All cocreators contributed to the PRODUCES (problem, objective, design, end users, cocreators, evaluation, and scalability) statement (Textbox 1). The reporting framework covers planning, conducting, process components, procedure methodologies, and evaluation (Table 2). Figure 1 depicts the logic model for performing the cocreation process, which was derived from the protocol format by Leask et al [27] and the Medical Research Council framework for designing complex interventions [29].

**Study objectives and methodology using the PRODUCES (problem, objective, design, end users, cocreators, evaluation, and scalability) framework, based on principles for cocreating health interventions developed by Leask et al [27].**

- Problem: in Islamabad, Pakistan, there is a lack of community readiness to implement school-based sexuality education.
- Objective: to create digital content that will promote, raise community awareness, and prepare students for sexuality education in schools. Partner with social media influencers to explore online digital platforms for health promotion (influencer marketing).
- Design: participatory action research
- End users: community influencers, gatekeepers, parents, teachers
- Cocreators: researchers, social media influencers, parents, teachers, health department representatives, community leaders
- Evaluation: process evaluation and performance evaluation (Table 2)
- Scalability and dissemination: generalizable model

**Table 1:** Comprehensive reporting framework for cocreation of complex health interventions covering planning, conducting, process components, procedure methodologies, and evaluation as developed by Leask et al [27].

Reporting framework		Description
**Planning**		
	PRODUCES^a^ framework, how was the aim of the study framed?	Textbox 1 presents the aim of the study using the PRODUCES framework.
	Explain the criteria used for sampling	Purposive convenience sampling was used to recruit the cocreators specified in the PRODUCES framework.
	In what setting did the sampling occur?	Cocreators were recruited from CRA^b^ participants and snowballing.
	How many individuals engaged as cocreators?	4 to 5 researchers, 5 community stakeholders, and 3 to 4 social media influencers.
	Describe the cocreators, such as their demographics, etc.	Presented in the Results section.
**Conducting**		
	Explain the methods used to manifest ownership	All cocreators were offered the chance to join the group and participate in the intervention’s development. Their time and efforts in the distribution of information were acknowledged.
**Procedure components**		
	What level of participation was there from the cocreators?	Equal participation by all cocreators
	How was the overall aim presented?	Each panel discussion and workshop began with a summary of the process’s aims and objectives.
	How was the purpose of each meeting presented?	Presented at the start of each discussion
	What were the rules and responsibilities of participation agreed upon?	Cocreators were informed about their right to equal participation and the opportunity to contribute ideas for intervention development.
**Procedure methods**		
	In which areas did cocreators require upskilling	Nonacademic cocreators needed to learn more about research methodologies and health promotion. Researchers improved their knowledge of collaborating with digital influencers to promote health. For this purpose, presentations were given during the FGDs^c^.
	What previous evidence was reviewed and how?	The CRA findings and the status of sexuality education in Pakistan, Pakistan’s reproductive and sexual health statistics, how CSE^d^ may promote health, and how CSE in schools is beneficial for adolescents were reviewed.
	If a prototype was developed, describe the prototype and the prototyping process	Following cocreators’ feedback, a prototype or video content was developed and further refined after the second FGD.
	Describe the frequency and duration of meetings	Two FGDs of approximately 2 hours each
	Give examples of interactive techniques or methods used	Brainstorming sessions and presentations at the start of each session. Open discussion sessions and continual feedback from the participants.
	Give examples of fieldwork techniques or methods used	Testing without the participation of the end user, with only the involvement of the cocreators
	Give examples of how iteration occurred during the process	Following discussions with cocreators, a prototype was developed and polished after the second FGD.
**Evaluation process**		
	Explain how cocreator satisfaction and contribution were evaluated, for example, reporting on attendance rates, questionnaires, and interviews	A detailed process evaluation was conducted with post-FGD feedback regarding the session gathered on the web from the cocreators.
	How are results reported back to stakeholders and the public?	Presentations were shared at the beginning of each session. Moreover, any future publications and reports will be shared with the cocreators.

^a^PRODUCES: problem, objective, design, end users, cocreators, evaluation, and scalability.

^b^CRA: community readiness assessment.

^c^FGD: focus group discussion.

^d^CSE: comprehensive sexuality education.

**Table 2:** Framework for evaluating the performance of health promotion content on social media, adapted from the framework proposed by Neiger et al [30] with metrics and indicators for video evaluation.

Key performance indicators	Definition	Metrics
Exposure	The number of times content is viewed on social media.	Number of views
Engagement	The number of people who participate in creating, sharing, and using the content.	Likes, comments, shares, engagement per 100 views, and reactions (emoji reactions)
Reach	The number of people who have contact with social media content.	Number of people reached
Audience demographics	Data on audience-specific factors such as age, gender, and geographic location.	Age, gender, and geographic location of the audience
Audience retention	The duration of the video that was watched by the audience.	Average seconds audience watched the video, average percentage of video watched by the audience
Insights	Audience feedback from social media platforms	Sentiment analysis of audience feedback, qualitative content analysis of the audience feedback

### Cocreators and Recruitment

Purposive convenience sampling was used to identify and recruit potential participants for this study. Individuals, who took part in a web-based survey conducted in Islamabad on CSE, were asked whether they were interested in participating in this study, and those who said yes were sent an invitation [20]. Snowballing was also used to recruit and identify additional participants. Researchers or academicians, parents, teachers, Pakistani health department personnel, nongovernmental organization representatives, and social media influencers were among the cocreators.

### Intervention Development and Focus Group Discussions

In January 2021, an online focus group discussion (FGD) was conducted to discuss awareness messages while considering the local context. The discussion focused on the content of CSE, as well as misconceptions that needed to be addressed in the intervention, wording, and language, and possible online platforms for intervention dissemination. Collaborating with a social media influencer and leveraging established channels and pages on social media platforms was considered a strategic approach. Influencers already have a dedicated and engaged following, which may significantly boost the video’s visibility. By leveraging influencer marketing, the approach might increase the video’s reach, effectively targeting a broader and more relevant audience, and increasing both engagement and potential impact. On the basis of the conclusions of the initial FGD, video content was developed in collaboration with a social media influencer [31,32]. The second FGD with the cocreators was held in June 2021 and all cocreators were given the opportunity to analyze and critique the video content, resulting in post hoc refinement of the video’s content. The 2 videos were posted on social media on June 30, 2021, and July 3, 2021. Multimedia Appendix 1 contains information about the videos and links to view them. A professional transcribing service transcribed the FGDs. The data from the FGDs was analyzed using qualitative content analysis. The qualitative data were analyzed, and a coding system was developed by 2 independent researchers. To develop the initial coding system, an open coding approach was combined with an inductive manifest analysis [33]. MAXQDA Analytics Pro 2020 (VERBI Software) was used to analyze and maintain the qualitative data. The coding scheme and code overview are included in Multimedia Appendix 1.

### Evaluation Framework

The feasibility and piloting phases include evaluating the expected performance of the intervention, as well as testing procedures for process evaluation of the development of intervention [27,29]. Although a pilot study does not need to be a scaled-up version of the mainstage evaluation, it should address the primary uncertainties that arise during the development and piloting phase [27,29]. For in-depth insights, a combination of qualitative and quantitative approaches would likely be necessary to assess participation barriers and engagement [27,29]. Hence, with feedback from the cocreators, we developed a complete evaluation framework for the process evaluation of the FGDs and a detailed performance evaluation of the intervention for this project as detailed below.

### Process Evaluation of Intervention Development

A process evaluation was carried out to assess the robustness of the participatory cocreation process and participant feedback regarding the sessions was collected. The participants’ postsession feedback was collected via a web-based survey. During the second FGD, the cocreators assessed the videos developed after the first FGD using the framework which was customized using the assessment criteria proposed by Kowatsch et al [34] for digital health interventions. Participants rated ease of use, content quality, esthetics, perceived effectiveness, perceived enjoyment, safety, negative connotations, and cultural context.

### Evaluation of Intervention Performance

The performance indicators and metrics used to evaluate the videos were adapted from the framework for assessing health promotion interventions designed for social media by Neiger et al [30]. In the second FGD, social media influencers were also asked about metrics and performance indicators and how they assess the performance of their content. To evaluate the performance of the videos, information was acquired from social media sites (February 2022). The framework that was developed for evaluating the performance of health promotion content disseminated on social media is presented in Table 2. The detailed definitions and metrics used for the 4 performance indicators are included in Table 2.

### Sentiment Analysis and Qualitative Content Analysis of Audience Feedback

For the fourth key performance indicator, audience insights, we used sentiment analysis and qualitative content analysis on the received comments to evaluate the audience’s feedback to the videos. Sentiment analysis is a method of examining the opinions, sentiments, emotions, and attitudes represented in textual data [35-37]. As a consequence, it appears to be an intuitive approach of assessing received comments and feedback and determining a quantitative score for the associated sentiment [35-37]. We used a lexicon-based technique for sentiment analysis, which involves matching a lexicon of sentiment terms with the text under analysis. We used the Bing lexicon, which comprises of approximately 6800 words and was created over a long period of time for analyzing customer reviews, beginning in 1995 [35,36]. Each matching word in the Bing lexicon is assigned a positive or negative sentiment. After that, for each comment, the net sentiment is calculated by subtracting the number of negative words from the number of positive words. If the net sentiment of a comment is positive, zero, or negative, it is categorized as positive, neutral, or negative, respectively [35,36]. However, the Bing lexicon is for the English language, and because the videos’ audience was largely Pakistani, some of the comments were in Urdu. A native Urdu speaker reviewed and translated the comments to English. RStudio Version 1.3.1093 was used to perform the lexicon-based sentiment analysis.

Viewers’ comments were analyzed using qualitative content analysis to explore the audience’s emotions and feedback in greater depth [33]. Using the National Research Council of Canada word emotion lexicon classification, an intuitive coding scheme was developed [38]. Fear, joy, trust, anger, sadness, disgust, anticipation, and surprise were all included in the coding scheme, as were positive and negative sentiments. Other codes, such as curses and suggestions, were also added to the coding system. The reasons behind the sentiments and emotions associated with the audience feedback were investigated qualitatively in depth. For rigor, 2 researchers independently reviewed the coding system and data. MAXQDA Analytics Pro 2020 was used to analyze and maintain the comments or feedback. The coding scheme and code overview are included in Multimedia Appendix 1.

### Ethical Considerations

This study adhered to ethical standards for research involving human participants and was approved by the National Bioethics Committee of the Pakistan Health Research Council (4-87/NBC-453/20/1815). All procedures followed the ethical guidelines of Pakistan Health Research Council. Informed consent was obtained from all participants in the FGDs, with participants being informed that their involvement was voluntary, anonymous, and that they could withdraw at any time. Consent was recorded electronically. To ensure privacy and confidentiality, no personally identifiable information was linked to the focus group data, or any other data collected. Audience feedback and insights were gathered from publicly available online comments on videos. Because many commenters did not use their real names and it was not feasible to obtain consent from all viewers, only nonidentifiable data that was available publicly was collected. Participants in the FGDs did not receive compensation, as the study relied on voluntary participation. In addition, no images or visual materials containing identifiable participants were included in this manuscript or its supplementary materials.

## Results

### Participant Information

**Figure 2:**
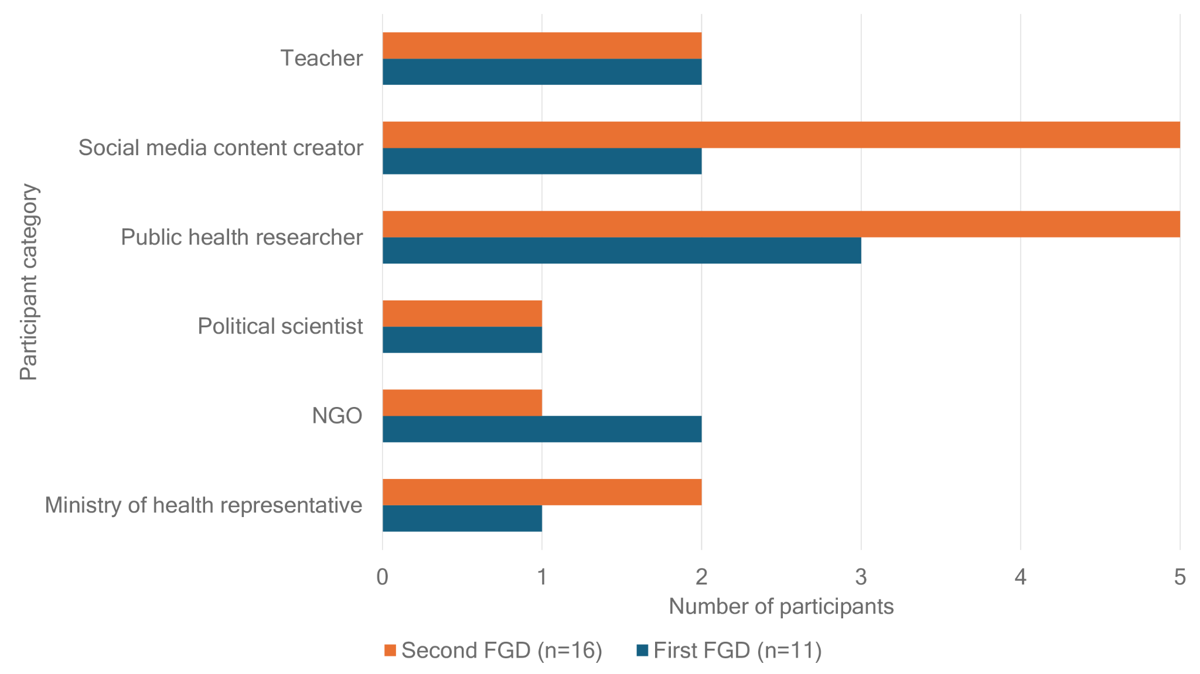
Background of the cocreators (participants) in the first and second focus group discussions (FGDs). NGO: nongovernmental organization.

Fifteen participants volunteered to take part in the first FGD, with 11 (73%) of them participating in the discussion session. Nineteen participants consented to take part in the second FGD, with 16 (84%) of them attending the FGD. The participants in the discussion sessions included public health experts, officials from the Ministry of Health, social media content creators, non-governmental organization representatives, political scientists, and teachers. Figure 2 depicts the precise composition of the FGD sessions.

### Intervention Development: First and Second FGD

Panelists recommended that the target audience should be narrowed down to influencers and gatekeepers. According to the panelists, one of the approaches being used to provide contraceptive education in Pakistan is storytelling. When producing video content, it was determined that targeting misunderstandings was crucial. Participants believed that community leaders, parents, teachers, and young adults, rather than the public, should be the target audience. On the basis of the panelists’ thoughts and opinions, it was decided to develop 2 videos, one focusing on what CSE is and the other on the misconceptions surrounding CSE in Pakistan. The first video provided information on CSE and its benefits, while the second addressed common misconceptions. Table 3 includes details on participant feedback regarding preferred approaches for CSE, the social media platforms recommended for video dissemination, and the intended target audiences for the developed videos.

**Table 3:** Participant feedback on preferred approaches for comprehensive sexuality education, targeted social media platforms for video dissemination, and intended audience for developed videos.

Category and item			Participants (n=5), n (%)
**Approaches**			
	Conservative religious approach	1 (20)	
	Human rights–based approach	0 (0)	
	An amalgamation of both	4 (80)	
**Social media platforms**			
	YouTube	4 (80)	
	Instagram	3 (60)	
	Facebook	4 (80)	
	Twitter	1 (20)	
	WhatsApp	1 (20)	
	Snapchat	1 (20)	
**Target audiences**			
	General public	0 (0)	
	Influencers	3 (60)	
	Young people	4 (80)	
	Teachers	4 (80)	
	Parents	3 (60)	

As there were some new participants, the second FGD began with a brief overview of CSE and a recap of the previous FGD. The PRODUCES Framework and an outline of the cocreation process were presented to the group. After that, the group was guided through the process of scripting, translating, and producing videos in collaboration with the social media influencer. Following that, the group was shown the 2 videos. After watching the videos, participants were asked to complete a feedback form evaluating the videos. The participants then debated the order in which the videos should be released, with some believing that the second video on misunderstandings should come out before the first. According to the participants, the first video had too many concepts, making it less engaging. It was also advised that the first video include some infographics to make it more interesting. Then, there was a discussion about how to evaluate the performance of the videos and what different metrics can be used to evaluate social media content. Some of the key indicators that social media influencers use to evaluate their content and how to interpret them were used to develop the framework for the evaluation of the health promotion content:

The content would be entirely different if it's targeting teachers or influencers, it would be entirely different if it's targeting the community...But the fact is that there is a clear-cut user interest in the topic.

Social media influencer, male

Targeting the stakeholder in the communities so religious leaders, policy makers and tell them with storytelling approaches, why it’s so important to do this education [CSE] and show them why this is necessary.

Public health researcher, male

Start from something that’s very relatable (violence and staying safe). And, I’d like to add that whatever components [CSE] that one decides to in the end reach to the target audience, it’s extremely important to make sure that it’s sensitized with the culture because if it’s in conflict with that, your every single approach that you’re going to try will invariably not end up getting you the result that you want. Start on topics that are generally more acceptable to the community, so I understand talking about maybe violence and safety and biological changes is the best way to go as a starting point.

Schoolteacher, female

I personally think it shouldn’t include data and numbers for mass market. It should be more human. It should be more localized.

Social media influencer, male

When we put our content on Facebook, because a lot of our content is insight driven. The intention is to gauge if it landed or not, through comments and shares, primarily, views, of course, and I’ll just move on to the problem with views. But before views, we look at comments and shares, because that is where we get to know if it connected with the audience or not. You’ll only share the content if you endorse it, because by sharing the content, and by putting it on your Facebook page, you’re saying, I approve of this message...By tagging other people in the comments, you mentioned there is a qualitative side to the comments as well of course because the more people get tagged, it widens the reach...The views are, and I would like to give an example from our content just to explain what I mean. We have a video on YouTube, it’s episode three of our web series. On YouTube, it has something around 150,000-160,000 views. On Facebook, it has reached around 2.8 million views organically...On YouTube, although 150,000 people are watching us, they’re watching us for if it was a seven-minute video, they have watched it for six minutes. On Facebook, if it was a seven-minute video, they would watch it for 40 seconds. So, I think view duration is another metric you should look at.

Social media influencer, female

### Evaluation of Intervention Development Phase: Process Evaluation

During the FGDs, cocreators shared their comments and suggestions on the intervention development, which were documented using a web-based questionnaire. During the second FGD cocreators evaluated the videos based on the following:

- Whether the content is culturally sensitive
- Any negative anticipated impacts
- The extent to which the use is safe with respect to any unintended side effects
- How engaging the video is
- The degree to which a person believes that the video improves their behavior regarding CSE
- The degree to which the interface, design elements, colors, and fonts are consistent and aesthetically pleasant
- The degree to which the content is accurate, relevant, and consistent
- The degree to which effort is required to take advantage of the digital content

**Figure 3:**
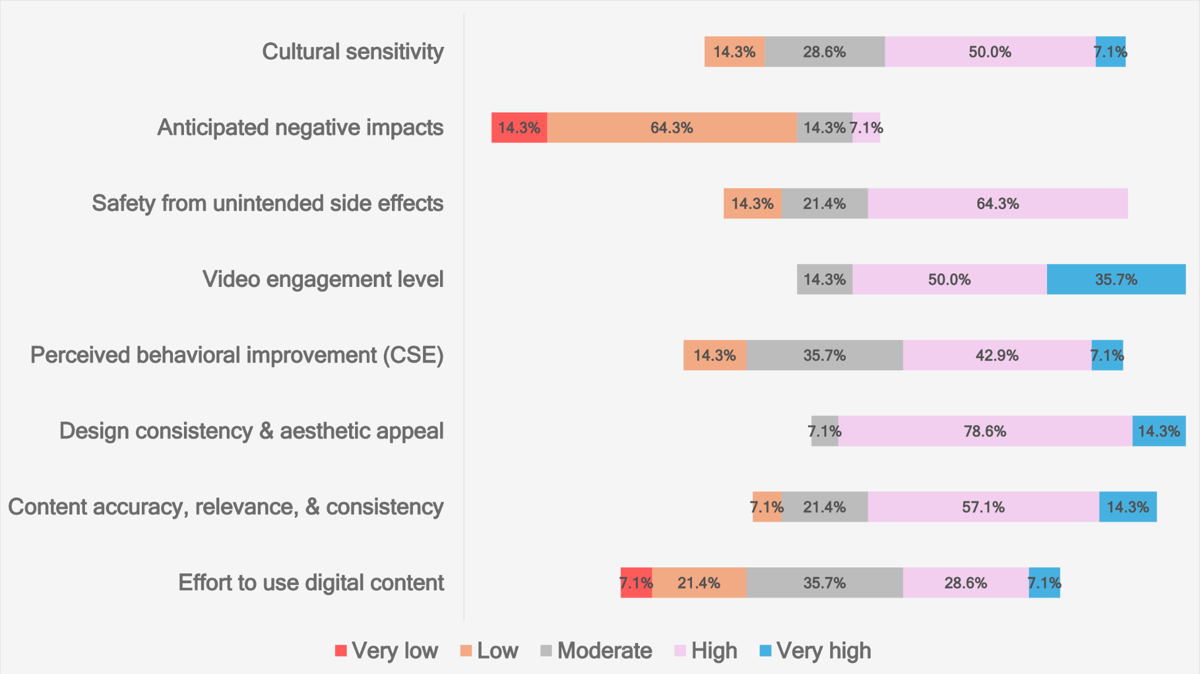
Diverging stacked bar chart of Likert scale ratings of cocreators for videos on culture, negative associations, safety, enjoyment, effectiveness, esthetics, content quality, and ease of use (n=14). CSE: comprehensive sexuality education.

Figure 3 presents the results of the cocreators’ video evaluation as a diverging stacked bar chart for the Likert scale, ranging from very low to very high.

**Figure 4:**
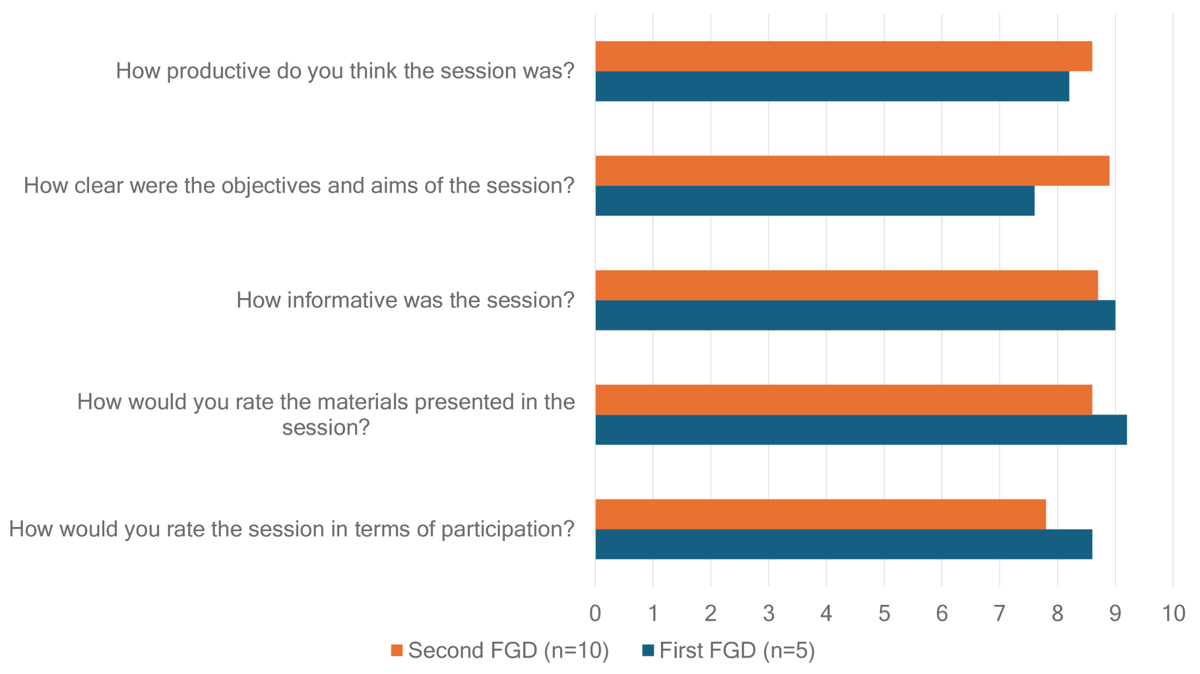
Feedback from first and second focus group discussions (FGDs; on a scale of 1 to 10, with 1 being the lowest and 10 being the highest) on cocreation session quality, clarity, participation, and materials by participants (first FGD n=5; second FGD n=10).

A detailed feedback form was developed to evaluate the FGDs, and the participants were encouraged to offer input about participation, materials presented, how informative the session was, how clear the aims and objectives were, and how productive the session was, which the participants filled out on the web after the session. In addition, the participants were asked to characterize the session using 3 adjectives. The frequency of the words was used to generate a word cloud for these adjectives. Figure 4 illustrates the average FGD feedback rating score on a scale of 1 to 10.

### Performance Evaluation

#### Audience Exposure, Engagement, and Reach

The data obtained from social media platforms were used to analyze video exposure, engagement, and reach (evaluation time period: July 2021-February 2022). Table 4 shows the information on audience exposure, engagement, and reach. According to the statistics, the videos were seen the most on Facebook (Meta Platforms) and the least on YouTube (Google LLC). However, views on Facebook and Instagram (Meta Platforms) require a 3-second view duration, whereas YouTube requires a 30-second view duration. For the first video, Instagram had the most audience engagement in terms of numbers, while YouTube had the lowest. For the second video, the highest levels of engagement were seen on Facebook. When the number of views was considered, Instagram had the most engagement per 100 views, followed by YouTube, and Facebook had the lowest. The percentage of likes versus dislikes for the first and second videos on YouTube was 100% and 99.5%, respectively. Table 4 shows the number of likes, comments, shares, and reactions by the audience to the videos.

**Table 4:** Analysis of audience engagement, exposure, and reach of health promotion videos across social media platforms (July 2021-February 2022).

Social media platform and metrics				First video		Second video
**Instagram**						
	Exposure or number of views (views at 3-second duration), n		17,486		10,035	
	Likes, n		1705		1668	
	Comments, n		38		37	
	Shares, n		590		740	
	Saves to collection, n		414		495	
	Engagement (sum of likes, comments, shares, saves to collection), n		2747		2940	
	Engagement per 100 views		15.71		29.30	
	Reach (number of people reached by the video), n		97,457		59,265	
**YouTube**						
	Exposure or number of views (views at 30-second duration), n		1422		4017	
	Likes versus dislike (%)		100		99.5	
	Likes, n		175		363	
	Comments, n		18		35	
	Engagement (sum of likes and comments), n		193		398	
	Engagement per 100 views		13.57		9.91	
	Reach (number of people reached by the video), n		20,100		98,900	
**Facebook**						
	Exposure or number of views (views at 3-second duration), n		169,600		317,300	
	Shares, n		178		264	
	Comments, n		70		152	
	**Reactions, n (%)**		1800 (100)		2900 (100)	
		Likes	1300 (72.22)		2300 (79.31)	
		Laugh	150 (8.33)		72 (2.48)	
		Heart	313 (17.39)		493 (17)	
		Wow	9 (0.5)		8 (0.28)	
		Sad	0 (0)		6 (0.21)	
	Engagement (sum of shares, comments, and reactions), n		2000		3300	
	Engagement per 100 views		1.18		1.04	
	Reach (number of people reached by the video), n		314,900		577,398	

#### Audience Demographics and Regional Scope

**Figure 5:**
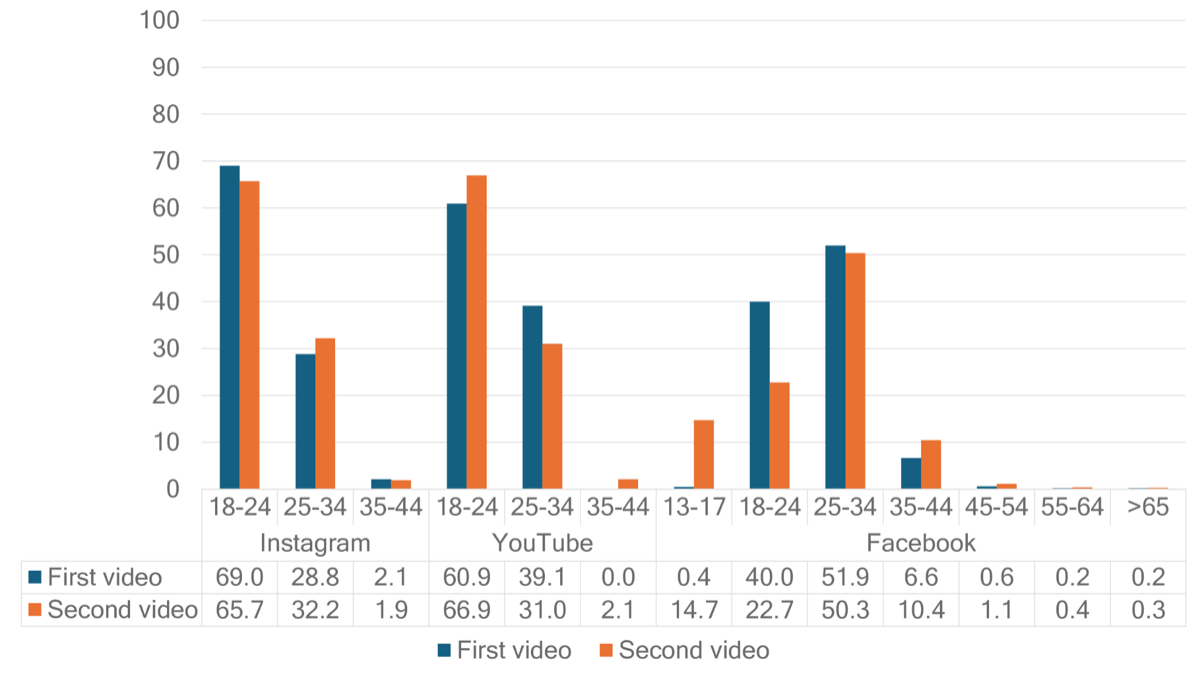
Age distribution as a percentage of total views for the first and second videos.

Figure 5 depicts the age groups of the audience for YouTube, Instagram, and Facebook. The 18-to-24-year age group had the most views on YouTube and Instagram, while the 25-to-34-year age group had the most views on Facebook. The viewers of the 13-to-17-year age group were only reached on Facebook.

Table 5 shows the gender distribution and geographic area of the Pakistani audience. Gender distributions were comparable across all platforms, with men watching the videos more than women. Instagram had the highest percentage of female viewers. Although there was no regional data available for YouTube, nearly half of all views on YouTube were from Pakistan. Table 5 shows the provincial distribution of Instagram and Facebook audiences. Punjab and Sindh provinces had the largest percentage of viewers for both platforms. Other nations accounted for 5942 and 13,005 Facebook views, constituting 3.5% (5942/169,600) and 4.09% (13,005/317,300) of the views, of the first and second video, respectively, which are shown by a heat map in Multimedia Appendix 1. These data were only available for the Facebook audience.

**Table 5:** Gender distribution, geographic location, and retention metrics of the audience for the Videos across social media platforms (July 2021-February 2022).

Platform				First video		Second video
**Gender (%)**						
	**Instagram** **(Meta Platforms)**					
		Male	82.2		80.7	
		Female	17.8		19.3	
	**YouTube** **(Google LLC)**					
		Male	83.5		90.1	
		Female	16.5		9.9	
	**Facebook** **(Meta Platforms)**					
		Male	91		83	
		Female	9		17	
**Locations (%)**						
	**Instagram**					
		Punjab	51.9		52.7	
		Sindh	33.2		31.6	
		Islamabad	6.9		7.1	
		Khyber Pakhtunkhwa	5.0		5.5	
		Baluchistan	1.6		1.4	
	**Facebook**					
		Punjab	44.3		48.4	
		Sindh	30.0		26.1	
		Khyber Pakhtunkhwa	13.9		14.9	
		Baluchistan	4.6		4.4	
		Islamabad	4.3		3.7	
		Asad Kashmir	1.8		1.6	
		Gilgit Baltistan	1.1		0.9	
	**YouTube**					
		Pakistan	47.8		56.6	
**Audience retention metrics**						
	**Instagram**					
		Average time audience watched the video (seconds)	10		30	
		Proportion of videos watched by the audience (%)	3		7	
	**YouTube**					
		Average time audience watched the video (seconds)	151 seconds		163 seconds	
		Proportion of videos watched by the audience (%)	45		38	
	**Facebook**					
		Average time audience watched the video (seconds)	11 seconds		13 seconds	
		Proportion of videos watched by the audience (%)	3		3	

#### Audience Retention

The statistics on audience retention are included in Table 5. The audience retention for the first video on Instagram was 3%, whereas it was 7% for the second video. Audience retention on Facebook was similarly low, at only 3% for both the first and second videos. Although the videos on YouTube had the least views, they had the most audience retention, with 45% and 38% for the first and second videos, respectively.

#### Insights: Audience Feedback Sentiment Analysis

Audiences on Facebook, Instagram, and YouTube engaged with the videos in a variety of ways, including comments, tags, and likes. The videos received a total of 222 comments across the 3 social media platforms, including comments where only other individuals were tagged and only emoticons were used in the comments, with 64 (28.8%) comments on episode 1 and 158 (71.2%) comments on episode 2. Following the removal of comments with just tags and emoticons, we were left with 187 (84.2%) comments: 121 (54.5%) on Facebook, 19 (8.6%) on Instagram, and 47 (21.2%) on YouTube. Of the 126 comments that matched the Bing lexicon, 83 (37.4%) were on Facebook, 14 (6.3%) on Instagram, and 29 (13.1%) on YouTube. Table 6 shows the descriptive statistics of the net sentiment; overall, by social media platform, and by episode. On the basis of the net-sentiment values of all the comments, an overall mean value of 0.83 was calculated, indicating that the general sentiment of the audience feedback was positive. Depending on the social media platform, the most positive sentiment was expressed on YouTube (sentiment score 1.55), Instagram (sentiment score 0.71), and the least on Facebook (sentiment score 0.59). Positive sentiment feedback with mean scores of 0.86 and 0.81 were seen for the first and second episodes, respectively (Table 6).

**Table 6:** Descriptive statistics of net sentiment from Bing lexicon–based analysis of audience comments on Facebook, Instagram, and YouTube (July 2021-February 2022).

	Observations, n	Sentiment score, mean (SD)	Minimum	Maximum
Overall comments (both videos on all platforms)	126	0.83 (1.46)	–3	5
First video comments (on all platforms)	36	0.86 (1.31)	–2	4
Second video comments (on all platforms)	90	0.81 (1.53)	–3	5
Facebook comments	83	0.59 (1.55)	–3	4
Instagram comments	14	0.71 (0.83)	–1	2
YouTube comments	29	1.55 (1.24)	–1	5

#### Insights: Qualitative Content Analysis of Audience Feedback

Most of the positive feedback was complimentary in tone, with most of the remarks containing expressions of joy and trust in the content. Some teachers who commented on the videos said it was an important issue to discuss and that teaching such themes in class had been difficult for them. Some of the key reasons that the audience felt joy and trust while giving positive feedback, were that the videos were interesting, increased awareness, developed counternarrative, and considered cultural context and considerations, along with having quality content, quality screenplay, and good production quality.

Much of the negative feedback and remarks had an emotional connotation of anger, fear, and disgust. The audience did not agree with the content and or relevance of CSE. Some of the audience’s harsh comments included profanity and insults. These included anything from profanity to threats of rape. Some of the proposed alternatives suggested by the audience who not agreeing with the content included teaching children about sexuality education at home, as well as teaching religion and a path of abstinence. Some viewers also believed and feared that supporting CSE would simply promote obscenity and contaminate their children’s minds. They also felt that this was Western propaganda, which would harm society and have a detrimental impact. Another concern expressed in most of the negative comments was that children might initiate sex at a younger age because of CSE. In this regard, similarities were drawn between CSE being introduced in the West and children having their initial sexual encounter at a young age.

There were some suggestions for refining the video’s editing and delivering the topic in greater depth. Some people even asked that this series be continued because of its informative nature. Most of the recommendations were to educate children’s religious principles and abstinence as a means of achieving a better outcome than CSE. Some others expressed surprise as to why this issue was being propagated on social media, while others offered religious texts as a counternarrative set in a religious context. Some viewers were also pleasantly surprised because the subject was so different from what they had seen before, and they enjoyed the fact that this topic was being discussed. Some viewers seemed to believe that this is the appropriate route to follow and that things will improve in the future for the betterment of society. Some feared the future and questioned if CSE was the correct route to follow and whether implementing it in schools would have any beneficial outcomes.

## Discussion

### Principal Findings

The goal of this study was to use the cocreation process of complex health intervention development to develop, pilot, and evaluate the performance of an intervention, based on community readiness level; to respond to community resistance by introducing CSE content by considering its anticipated benefits; and address prevalent misconceptions via awareness and promotion content for digital platforms. According to the findings of this study, social media platforms provide an opportunity to communicate, promote, and engage with key stakeholders for raising awareness and gaining support regarding CSE.

### Potential of Social Media for Health Promotion

Online social media platforms, which have an ever-increasing number of active users worldwide, have the potential to be effective vectors for health promotion, including sexual health [22,23]. The evidence supporting the positive impacts of social media interventions in enhancing sexual health is emerging, but there is still a scarcity of research that explicitly analyzes theoretical frameworks and uses rigorous study procedures [22,23]. Also, there is a lack of primary research on the applications, advantages, and limits of social media for health promotion and stakeholder communication [22,23]. The methods and outcomes of this study attempted to fill a critical gap in the literature in this aspect [22,23]. Although this was an exploratory pilot study to see how social media platforms could be used to raise awareness about CSE, the findings show that these forums can be effective tools for engaging and reaching a large audience for sexual health promotion, particularly in conservative settings like Pakistan. In comparison to previous similar research, in terms of exposure or views, 2 Australian studies that used YouTube videos to promote sexual health received 5300 [39] and 30,000 [40] views, respectively [23]. Similarly, an Irish study used a YouTube video for abortion awareness that had >75,000 views [23,41]. This highlights that using social media platforms and collaborating with social media influencers for health promotion and communication has a significant advantage, including improved interactions and engagement, improved information dissemination, increased access to health information, and the ability to create counter narratives for stigmatized health issues.

### Platform-Specific Insights

The videos were disseminated on a variety of social media platforms, allowing a comparison of which content would perform optimally on which platform. Since the videos were more than 5 minutes in length, and thus deemed lengthy, viewer retention was highest on YouTube. There was also a substantial difference in retention on YouTube versus Facebook and Instagram. This suggests that retention for lengthier content is considerably higher on YouTube, whereas shorter content may perform better on Facebook and Instagram. Facebook is also one of the most popular sites for sharing health-related content [23,30]. In terms of reach, it was found that Facebook had the largest reach, suggesting that Facebook might be the best medium for reaching a broader audience. This can also be seen on Facebook when looking at demographics, audience reach, and exposure to content. When it came to audience distribution throughout the world, Facebook had a greater reach. Unfortunately, there were no geographic data available for the YouTube audience to compare to other platforms.

### Audience Sentiment and Feedback

Most of the audience had a positive sentiment toward the developed content, according to audience feedback and sentiment analysis. Sentiment analysis of audience response gives a quantitative estimate of the overall sentiment of the feedback [35]. Although it gives an overall numerical representation, it fails to reflect the complexities of the audience’s motives and perspectives [35]. A qualitative content analysis was undertaken to better grasp the deeper reasoning and emotions underlying the viewers’ responses. Much of the negative response was attributable to widespread societal preconceptions about CSE, which were conveyed as negative feedback and occasionally as profanities and insults. Given the topic at hand, this was anticipated. However, careful scripting and consideration of cultural sensitivity throughout the intervention development phase resulted in a more sympathetic approach to the subject. Positive responses also demonstrated that the audience understood the importance of the topic, with most comments expressing joy, confidence, and trust in the content.

### Limitations of Social Media for Health Promotion

Most of the limitations of using social media platforms for health promotion are related to quality concerns, as well as a lack of reliability, confidentiality, quality monitoring, and privacy [22,23]. Keeping these issues in mind, a collaborative cocreation approach provided transparency and a mechanism for attempting to address these concerns. Cocreation is a method for generating complex health interventions that has been found to increase uptake and user engagement. Engaging a diverse range of stakeholders, particularly social media influencers, throughout the intervention development phase provided in-depth viewpoints, particularly for developing a health promotion intervention on social media platforms. The FGDs also allowed participants to explore topics such as content, cultural sensitivity, influencer marketing, and the development of a performance evaluation framework. Adapting the evaluation framework developed by Neiger et al [30] provided a foundation for assessing the performance of the videos. The use of mixed method approaches in the evaluation framework, particularly sentiment analysis and qualitative content analysis, allowed a more in-depth understanding of the audience feedback. Other key performance indicators and metrics give a more comprehensive quantitative approach to assessing the performance of content shared on social media platforms. However, the results of the pilot study should be interpreted with caution when planning to scale it up [29]. When the intervention is scaled up, its impact may be less or more varied, and response rates may be lower [29]. While bearing in mind that the efficacy of the intervention was not evaluated during the piloting stage, and only a process and performance evaluation was conducted, the feasibility of the intervention is one of the major limitations of the study. Despite this, the comprehensive evaluation framework and intervention development phase tried to address many of the uncertainties that may occur during scaling up, offering rich data and deeper insights into user engagement, audience feedback, and stakeholder perspectives.

Overall, the findings of the process and performance evaluation show that confronting such culturally sensitive topics with careful stakeholder participation and novel ways of health intervention development may be a path forward in addressing stigmatized health issues. Digital health solutions and interventions also provide an opportunity for health communication and promotion in settings such as Pakistan, where people are increasingly using digital and social media platforms [26]. The findings are equally encouraging, and it can be stated that social media platforms are a strong and viable way of generating community support and engagement and raising awareness for supporting CSE when faced with resistance. Future research is needed to assess the efficacy of such interventions over time, particularly in terms of audience behavior and perception.

### Conclusions

This is one of the first studies to develop and evaluate a complex health intervention for CSE promotion on social media platforms using a cocreation methodology. The findings indicate that the audience is responsive to content developed for CSE promotion on social media platforms and that these platforms have a large demographic and overall beneficiary reach. End-user engagement was also favorable, and the audience response revealed the reasons for positive and negative criticism in detail. Future research is needed to assess the long-term efficacy of such scaled-up interventions, particularly regarding end-user behavior and perception changes.

## Appendix

Multimedia Appendix 1 Comprehensive overview of the intervention development process, including insights from 2 focus group discussions and the cocreation of educational video content to address misconceptions about comprehensive sexuality education. In addition, this appendix presents detailed coding results from qualitative analyses, audience feedback categorized by sentiment, and geographical heat maps of video reach, offering a holistic understanding of community readiness and engagement strategies tailored to the local cultural context.

## Abbreviations

CSE: comprehensive sexuality education
FGD: focus group discussion
PRODUCES: problem, objective, design, end users, cocreators, evaluation, and scalability
SDG: sustainable development goal
SRH: sexual and reproductive health
UNESCO: United Nations Educational, Scientific, and Cultural Organization

## Data Availability

The datasets generated during and analyzed during this study are available from the corresponding author on reasonable request.
