# Supplementary material for: Promoting Comprehensive Sexuality Education in Pakistan Using a Cocreated Social Media Intervention: Development and Pilot Testing Study
Source: JMIR Form Res. 2024 Dec 20;8:e52651. doi: 10.2196/52651 (PMC11699495; doi:10.2196/52651)

# Focus Group Discussions and Intervention Development Phase

An online focus group discussion (FGD) was conducted in January 2021 to explore awareness messages while considering the local context. The discussion revolved around the content of Comprehensive Sexuality Education (CSE), addressing common misconceptions, and identifying suitable internet platforms for disseminating the intervention. Based on insights from this first focus group, video content was co-developed with a social media influencer. In June 2021, a second FGD was held with the co-creators, who were invited to review and critique the video content, leading to further refinements. The FGDs were professionally transcribed, and the data was analyzed using qualitative content analysis.

## Video Content Overview

The two videos developed from this process shed light on the ongoing debate surrounding CSE in educational settings.

### Video 1:

The first video depicts an active classroom where a determined teacher explains the importance of CSE to a group of students. Through a well-structured discussion, the teacher addresses common misconceptions about discussing sexuality with youth. Key components of the CSE curriculum, such as gender equality, puberty education, reproductive health, and STI prevention, are highlighted, emphasizing the role of CSE in informed decision-making.

The scenario takes a dramatic turn with the unexpected arrival of law enforcement, resulting in the teacher’s arrest by a police officer. This incident symbolizes societal concerns and resistance to CSE, reflecting broader tensions between education and authority. The conflict illustrates the diverse viewpoints within communities about the appropriateness of CSE in schools.

### Video 2:

The second video continues the narrative in a police station, where Inspector Irshad (Ali Gul Pir, Actor) interrogates the detained teacher on allegations of spreading "obscenities" to young minds. The dialogue goes into societal attitudes, cultural sensitivities, and policy implications related to CSE. Inspector Irshad’s probing questions, contrasted with the teacher’s passionate defense of CSE, represent the wider cultural debate on the subject.

Both videos highlight themes of responsibility, cultural relevance, and collaboration, crucial for implementing CSE initiatives. They emphasize the need to align CSE with cultural and religious norms, while respecting diverse perspectives and sensitivities. Moreover, the discourse calls for collaboration among parents, educators, religious leaders, and policymakers to create a supportive environment for CSE.

### Community Readiness and Awareness

The videos were scripted based on the community’s readiness to discuss CSE. A community readiness assessment revealed resistance and denial stages, needing careful awareness strategies that respect cultural norms and clear up misconceptions. The videos were shared on a well-established YouTube channel run by a general practitioner, with accompanying Facebook and Instagram pages promoting health awareness in the Pakistani context, targeting Urdu-speaking audiences. Ali Gul Pir, a well-known Pakistani rapper, and television actor played the role of the police officer. His involvement, along with the established platform, functioned as an influencer marketing strategy to increase reach and engagement.

### Video Links

**YouTube Channel**:

[Daktar Saab YouTube Channel](https://www.youtube.com/@DaktarSaab)

**Trailer**:

[Watch the Trailer](https://www.youtube.com/watch?v=vn8mmH8RWYo)

**Video 1**:

- [Facebook](https://www.facebook.com/TheDaktarSaab/videos/228270992288741)
- [Instagram](https://www.instagram.com/p/CQv4Fpegl5W/)
- [YouTube](https://www.youtube.com/watch?v=aOBPWy1cEm0&t=2s&ab_channel=DaktarSaab)

**Video 2**:

- [Facebook](https://www.facebook.com/TheDaktarSaab/videos/1379060902475869)
- [Instagram](https://www.instagram.com/p/CQ3peg-AJeJ/)
- [YouTube](https://www.youtube.com/watch?v=5WE90v1vcy0&t=7s&ab_channel=DaktarSaab)

OVERVIEW OF CODES (Focus group discussion sessions)

| COLOR | PARENT CODE | CODE | CODED SEGMENTS (ALL DOCUMENTS) | % CODED SEGMENT (ALL DOCUMENTS) |
| --- | --- | --- | --- | --- |
| ● | REVISED GUIDANCE | FOCUSES | 2 | 1.31 |
| ● | REVISED GUIDANCE | SUSTAINABLE DEVELOPMENT GOALS | 1 | 0.65 |
| ● | CSE | MISCONCEPTIONS ABOUT CSE | 1 | 0.65 |
| ● | CSE | RESEARCH EVIDENCE ON CSE | 1 | 0.65 |
| ● | CSE GUIDANCE | AIMS OF CSE | 2 | 1.31 |
| ● | CSE GUIDANCE | ORIGINAL GUIDANCE | 2 | 1.31 |
| ● | CSE GUIDANCE | REVISED GUIDANCE | 0 | 0.00 |
| ● | REVISED GUIDANCE | NEW ASPECTS | 1 | 0.65 |
| ● | MERGED CODING | INTRODUCTION OF THE GROUP | 2 | 1.31 |
| ● | MERGED CODING | CSE | 0 | 0.00 |
| ● | CSE | CSE GUIDANCE | 0 | 0.00 |
| ● | CSE GUIDANCE | DEFINITION OF CSE | 2 | 1.31 |
| ● | INFLUENCER-MARKETING | INLUENCER IN THE MEDICAL FIELD | 2 | 1.31 |
| ● | MERGED CODING | CONCLUSION | 2 | 1.31 |
| ● |  | MERGED CODING | 0 | 0.00 |
| ● | PLATFORMS | OWN PLATFORM | 1 | 0.65 |
| ● | DISSEMINATION | ADVERTISING STREAMS | 1 | 0.65 |
| ● | IMPLEMENTATION | MARKETING | 0 | 0.00 |
| ● | MARKETING | INFLUENCER-MARKETING | 1 | 0.65 |
| ● | DISSEMINATION | PLATFORMS | 1 | 0.65 |
| ● | PLATFORMS | FACEBOOK | 4 | 2.61 |
| ● | PLATFORMS | YOUTUBE | 1 | 0.65 |
| ● | PLATFORMS | AUDIENCE | 3 | 1.96 |
| ● | FEEDBACK | LANGUAGE | 1 | 0.65 |
| ● | LANGUAGE | EXPLANATION: LANGUAGE | 1 | 0.65 |
| ● | LANGUAGE | POSITIVE: DIALOGUE FORM | 1 | 0.65 |
| ● | IMPLEMENTATION | DISSEMINATION | 4 | 2.61 |
| ● | FEEDBACK | TOO MANY TOPICS/INFORMATION | 5 | 3.27 |
| ● | FEEDBACK | ADDITIONS | 2 | 1.31 |
| ● | FEEDBACK | MORE VISUAL/INTERACTIVE PRESENTATION | 2 | 1.31 |
| ● | FEEDBACK | VIDEO LENGTH | 3 | 1.96 |
| ● | FEEDBACK | SEXUAL VIOLENCE | 1 | 0.65 |
| ● | SEXUAL VIOLENCE | EXPLANATION: MALE PERPETRATOR | 2 | 1.31 |
| ● | SEXUAL VIOLENCE | GENDER-NEUTRAL PERPETRATOR | 2 | 1.31 |
| ● | FEEDBACK | CHANGE ORDER OF VIDEOS | 1 | 0.65 |
| ● | IMPLEMENTATION | SCRIPT | 2 | 1.31 |
| ● | IMPLEMENTATION | VIDEO 1: INTRODUCTION TO CSE | 7 | 4.58 |
| ● | IMPLEMENTATION | VIDEO 2: CLARIFICATION OF MISCONCEPTIONS/MYTHS | 2 | 1.31 |
| ● | IMPLEMENTATION | FEEDBACK | 0 | 0.00 |
| ● | INTERVENTION/PILOT | PAST UPSCALE EXAMPLES FROM PAKISTAN | 0 | 0.00 |
| ● | PAST UPSCALE EXAMPLES FROM PAKISTAN | PROBLEMS OF SIMILAR INTERVENTIONS | 1 | 0.65 |
| ● | PAST UPSCALE EXAMPLES FROM PAKISTAN | AAHNUNG & RUTGERS | 1 | 0.65 |
| ● | INTERVENTION/PILOT | IMPLEMENTATION | 0 | 0.00 |
| ● | USE OF STATISTICS/NUMBERS | ACCEPTANCE DEPENDS ON AUDIENCE | 1 | 0.65 |
| ● | USE OF STATISTICS/NUMBERS | VISUAL PRESENTATION OF DATA | 2 | 1.31 |
| ● | USE OF STATISTICS/NUMBERS | REFUSAL TO USE DATA | 3 | 1.96 |
| ● | USE OF STATISTICS/NUMBERS | NEGATIVE RESPONSES | 1 | 0.65 |
| ● | APPROACHES | HOLISTIC APPROACH | 1 | 0.65 |
| ● | INTERVENTION/PILOT | ROLE OF SOCIAL MEDIA | 2 | 1.31 |
| ● | INTERVENTION/PILOT | FOCUS ON MAIN ASPECTS | 2 | 1.31 |
| ● | INTERVENTION/PILOT | USE OF STATISTICS/NUMBERS | 1 | 0.65 |
| ● | CONSIDERATIONS ABOUT THE CONTENT | APPROACHES | 0 | 0.00 |
| ● | APPROACHES | SYSTEMIC APPROCH | 1 | 0.65 |
| ● | APPROACHES | HUMAN RIGHTS-BASED APPROACH | 4 | 2.61 |
| ● | APPROACHES | RELIGIOUS-BASED APPROACH | 5 | 3.27 |
| ● | FIRST TOPIC (CONSIDERATIONS/SUGGESTIONS) | UNDERSTANDING/DEVELOPMENT OF THE HUMAN BODY | 3 | 1.96 |
| ● | CONSIDERATIONS ABOUT THE CONTENT | STORYTELLING | 6 | 3.92 |
| ● | STORYTELLING | BENEFITS | 1 | 0.65 |
| ● | CONSIDERATIONS ABOUT THE CONTENT | DESENSITIZATION | 1 | 0.65 |
| ● | PROPER ACCESS TO THE CONTENT | CREATE A FEELING OF BELONGING TO A GROUP | 2 | 1.31 |
| ● | CONSIDERATIONS ABOUT THE CONTENT | FIRST TOPIC (CONSIDERATIONS/SUGGESTIONS) | 1 | 0.65 |
| ● | FIRST TOPIC (CONSIDERATIONS/SUGGESTIONS) | VIOLENCE | 3 | 1.96 |
| ● | FIRST TOPIC (CONSIDERATIONS/SUGGESTIONS) | SAFETY | 2 | 1.31 |
| ● | CONSIDERATIONS ABOUT THE CONTENT | VIEWER | 1 | 0.65 |
| ● | CONSIDERATIONS ABOUT THE CONTENT | ACCESS TO THE INTERNET | 1 | 0.65 |
| ● | CONSIDERATIONS ABOUT THE CONTENT | PROPER ACCESS TO THE CONTENT | 2 | 1.31 |
| ● | PROPER ACCESS TO THE CONTENT | CUSTOMIZED CONTENT TO THE TARGET GROUP | 3 | 1.96 |
| ● | TARGET GROUP (CONSIDERATIONS) | STAKEHOLDER | 1 | 0.65 |
| ● | TARGET GROUP (CONSIDERATIONS) | FAMILIES/ADOLESCENTS | 1 | 0.65 |
| ● | INTERVENTION/PILOT | BASIC IDEA | 1 | 0.65 |
| ● | INTERVENTION/PILOT | CONSIDERATIONS ABOUT THE CONTENT | 2 | 1.31 |
| ● | MERGED CODING | INTERVENTION/PILOT | 0 | 0.00 |
| ● | INTERVENTION/PILOT | CO-CREATION | 2 | 1.31 |
| ● | INTERVENTION/PILOT | AIMS OF THE INTERVENTION | 6 | 3.92 |
| ● | INTERVENTION/PILOT | TARGET GROUP (CONSIDERATIONS) | 6 | 3.92 |
| ● | COMMUNITY READINESS ASSESSMENT | INTERVIEW PARTICIPANTS | 1 | 0.65 |
| ● | COMMUNITY READINESS ASSESSMENT | DEFINITION OF CSE | 1 | 0.65 |
| ● | COMMUNITY READINESS ASSESSMENT | RESULTS | 2 | 1.31 |
| ● | MERGED CODING | ISSUES AROUND ADOLESCENT HEALTH | 1 | 0.65 |
| ● | MERGED CODING | COMMUNITY READINESS ASSESSMENT | 0 | 0.00 |
| ● | COMMUNITY READINESS ASSESSMENT | AIM OF THE STUDY | 2 | 1.31 |
| ● | COMMUNITY READINESS ASSESSMENT | COMMUNITY READINESS MODEL | 2 | 1.31 |
| ● | COMMUNITY READINESS ASSESSMENT | METHODS | 1 | 0.65 |
| ● | MERGED CODING | CURRENT SITUATION IN PAKISTAN | 2 | 1.31 |
| ● | CURRENT SITUATION IN PAKISTAN | INTERNET USAGE IN PAKISTAN | 1 | 0.65 |
| ● | INTERNET USAGE IN PAKISTAN | SOCIAL MEDIA USERS | 1 | 0.65 |
| ● | CURRENT SITUATION IN PAKISTAN | RELIGIOUS OPPOSITION TO CSE | 3 | 1.96 |

OVERVIEW OF CODES (Audience feedback and comments)

| Color | Code | Coded segments (all documents) | % Coded segments (all documents) |
| --- | --- | --- | --- |
| ● | Disgust | 36 | 8.53 |
| ● | Sadness | 7 | 1.66 |
| ● | Negative | 46 | 10.90 |
| ● | Fear | 11 | 2.61 |
| ● | Positive | 101 | 23.93 |
| ● | Curse | 17 | 4.03 |
| ● | Surprise | 10 | 2.37 |
| ● | Anticipation | 6 | 1.42 |
| ● | Suggestion | 14 | 3.32 |
| ● | Joy | 81 | 19.19 |
| ● | Trust | 68 | 16.11 |
| ● | Anger | 25 | 5.92 |

Figure 1: Heat map of international audience by geographical location for first video on Facebook.


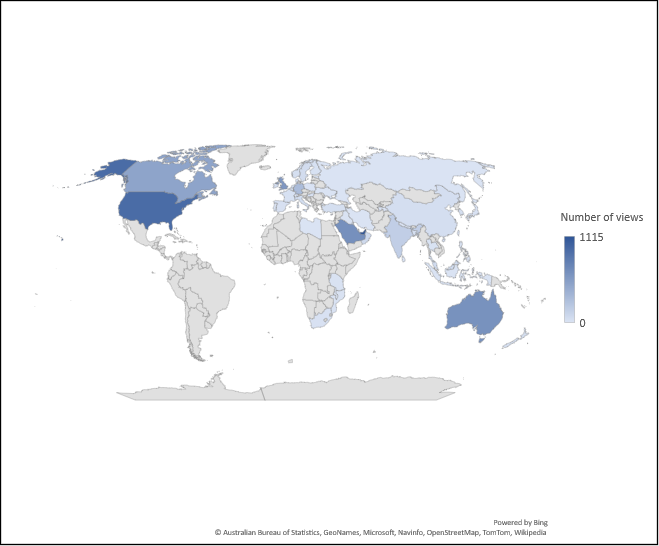


Figure 2: Heat map of international audience by geographical location for second video on Facebook.


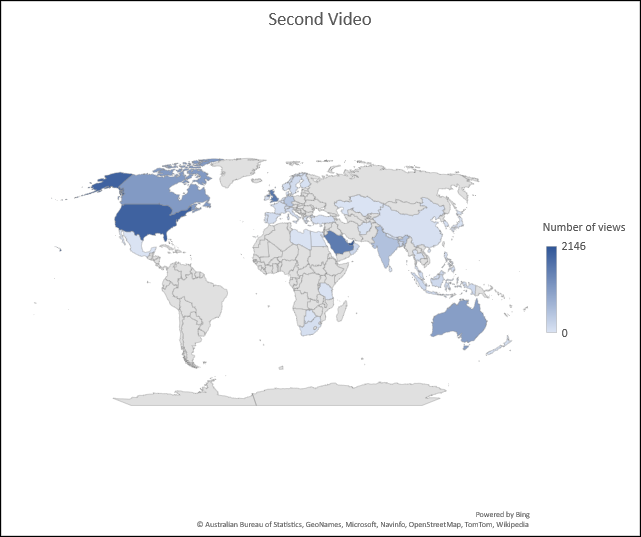

Supplement: Multimedia Appendix 1 [file formative_v8i1e52651_app1.docx]
